# Negative Emotional Content Disrupts the Coherence of Episodic Memories

**DOI:** 10.1037/xge0000356

**Published:** 2017-09-14

**Authors:** James A. Bisby, Aidan J. Horner, Daniel Bush, Neil Burgess

**Affiliations:** 1 Institute of Cognitive Neuroscience, Institute of Neurology, University College London; 2 Department of Psychology, University of York; 3 Institute of Cognitive Neuroscience, Institute of Neurology, University College London

**Keywords:** episodic memory, emotion, hippocampus, pattern completion, memory coherence

## Abstract

Events are thought to be stored in episodic memory as coherent representations, in which the constituent elements are bound together so that a cue can trigger reexperience of all elements via pattern completion. Negative emotional content can strongly influence memory, but opposing theories predict strengthening or weakening of memory coherence. Across a series of experiments, participants imagined a number of person-location-object events with half of the events including a negative element (e.g., an injured person), and memory was tested across all within event associations. We show that the presence of a negative element reduces memory for associations between event elements, including between neutral elements encoded after a negative element. The presence of a negative element reduces the coherence with which a multimodal event is remembered. Our results, supported by a computational model, suggest that coherent retrieval from neutral events is supported by pattern completion, but that negative content weakens associative encoding which impairs this process. Our findings have important implications for understanding the way traumatic events are encoded and support therapeutic strategies aimed at restoring associations between negative content and its surrounding context.

Episodic memories typically comprise complex events that include multiple elements such as the people we meet, the objects we interact with, and the locations in which those encounters take place. Their retrieval is characterized by a rich recollective experience in which all of the event’s constituent elements are brought to mind (Tulving, 1983). For this holistic episodic retrieval to occur, the elements that form an event must be bound together, allowing for their subsequent reinstatement. The hippocampus plays an essential role as a convergence zone, binding together the separate elements of an event (Cohen & Eichenbaum, 1993; Damasio, 1989; Davachi, 2006; Eichenbaum, Yonelinas, & Ranganath, 2007; O’Keefe & Nadel, 1978). Presentation of a partial cue will lead to the reinstatement of all event elements through a process of pattern completion in the hippocampus (Marr, 1971; McClelland, McNaughton, & O’Reilly, 1995; Norman & O’Reilly, 2003). While negative emotion clearly impacts memory for an event (Bradley, Greenwald, Petry, & Lang, 1992; Brown & Kulik, 1977; Cahill et al., 1996; Cahill & McGaugh, 1995; Christianson, 1992), the way in which it affects the binding of event elements into coherent memory representations remains controversial.

According to a “general facilitation” account, strong emotional content will strengthen all aspects of memory for the event, enhancing its availability at retrieval (McGaugh, 2003; Rubin, Boals, & Berntsen, 2008; Talarico, LaBar, & Rubin, 2004). In contrast, a ‘dual representation’ account argues that negative emotion will affect different aspects of memory in opposing ways (Brewin, Gregory, Lipton, & Burgess, 2010; Jacobs & Nadel, 1998): potentially enhancing memory for the negative content itself but weakening associations between the negative content and its context (i.e., other neutral aspects of the event). These two views therefore make competing predictions, one arguing for strengthened memory for the whole negative event, and the other for more fragmented memory due to impaired associations between elements.

When exposed to a negative experience, participants often report greater vividness, accuracy and confidence during recall (Cahill et al., 1996; Kensinger & Corkin, 2003), and show a recollection advantage for the negative details (Rimmele, Davachi, Petrov, Dougal, & Phelps, 2011; Sharot, Delgado, & Phelps, 2004; Sharot & Yonelinas, 2008). These findings are consistent with a general facilitation account, and might reflect boosting of hippocampal encoding or consolidation via fear-related processing in the amygdala (McGaugh, 2004). However, the mnemonic advantage seems specific to the negative items themselves, whereas memory for the associations between items or an item and its context is often impaired when negative items are present (Bisby & Burgess, 2014; Kensinger & Schacter, 2006; Madan, Caplan, Lau, & Fujiwara, 2012; Mather, 2007; Mather & Knight, 2008; Touryan, Marian, & Shimamura, 2007). According to the dual representation account, negative emotion down-modulates hippocampal processing, disrupting associative/relational binding, while amygdalar up-modulation facilitates encoding of the negative content of an event (Bisby, Horner, Horlyck, & Burgess, 2016; Jacobs & Nadel, 1998).

Recent studies have shown that our performance over multiple trials in retrieving different elements from the same event is statistically related, providing behavioral evidence that episodic memory reflects “coherent representations” supported by pattern completion (Horner & Burgess, 2013, 2014). In these studies, events involving triplets (person, object and location) were either encoded simultaneously or built up over a number of encoding trials in which all interelement associations were learned. A subsequent memory test of all associations quantified the holistic nature of episodic recollection in terms of the statistical dependency between retrievals from the same event—that is, when participants were successful in one cued retrieval from an event, they were more likely to be successful in other cued retrievals from the same event. This highlights the importance of binding together the elements of an event to form a coherent memory representation that can support holistic retrieval. In a further study, statistical dependency was found to be related to hippocampal activity and incidental reinstatement of all event elements in neocortical regions (Horner, Bisby, Bush, Lin, & Burgess, 2015).

We aimed to investigate the effects of negative emotion on the binding of different event elements into coherent mnemonic representations. Following previous studies (Horner et al., 2015; Horner & Burgess, 2013, 2014), we assessed the coherence of retrievals of within-event associations from multielement events involving neutral and negative elements. If negative emotion impairs associative binding, it should reduce the coherence of memories for negative events as evidenced by reduced statistical dependency between retrievals from the same event. If negative emotion strengthens all aspects of memory, including associations between elements, we should see increased coherence.

We performed three experiments in which participants were required to encode events comprising a person, location and object, each presented as images on screen. For half of the events, the person was a negative image, such as an injured individual. These events were either presented simultaneously as triplets (Experiment 1) or sequentially as overlapping pairs (Experiments 2 and 3) with participants instructed to imagine the elements interacting. Experiment 3 involved a 24hr delay between study and test to allow detection of any effects that might be supported by consolidation processes. Recognition memory was tested by cueing with a single element and asking whether the cue was old or new (recognition was only tested in Experiment 1). Associative memory for each pair of elements was assessed using a six-alternative forced choice with participants required to select the element that had been originally paired with the cue (Figure 1a). To explore potential neural mechanisms that might underpin the pattern of behavioral data, we constructed a simple computational model of associative learning (Marr, 1971).[Fig-anchor fig1]

## Experiment 1

### Method

#### Participants

A total of 17 participants (7 males) with a mean age of 23.36 years (*SD* = 3.88) were recruited from the university student population. A power analysis based on effect sizes reported in previous studies (Bisby et al., 2016; Bisby & Burgess, 2014) provided an approximate sample size required for each experiment (Experiment 1, *N* = 18; Experiments 2 and 3, *N* = 23; power = 0.80, α = .05). All experiments were approved by the University College London Ethics Committee and participants provided written informed consent prior to taking part in the study. Following test, participants were debriefed and paid.

#### Materials

**Figure 1 fig1:**
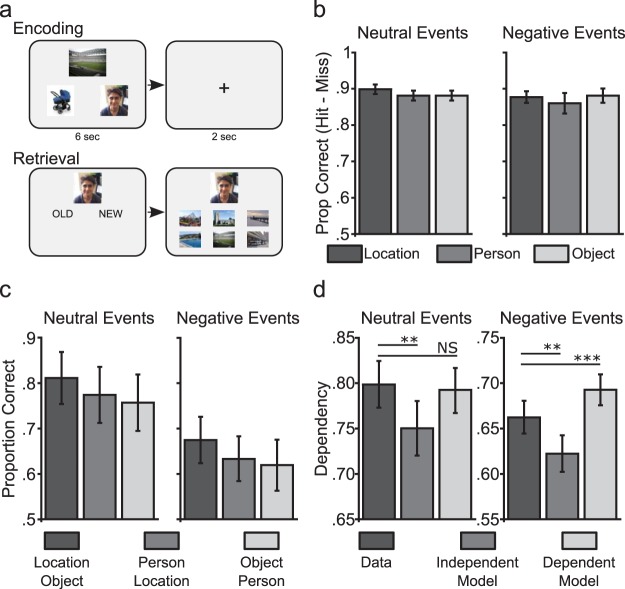
Memory for events encoded with simultaneously presented elements. (a) In Experiment 1, each encoding trial included three separate event elements (location, person and object) presented simultaneously and followed by a 2 s intertrial interval (ITI). At retrieval, a cue image was presented and participants were required to respond whether the image was old or new. If old, the participant was then presented with six options and instructed to select the image that had been originally presented with the cue image at encoding (see Methods). (b) Recognition accuracy for each element type (hits minus misses) was compared between neutral and negative events (collapsed across first and second presentation during test). (c) Associative memory performance for neutral and negative events split by the different element pair types for each event (collapsed across testing direction; chance performance = 0.17). (d) Dependency in the data was compared to independent and dependent models across neutral and negative events. Error bars represent standard error; NS = not significant. ** *p* = .001. *** *p* < .001.

Stimuli included a total of 216 images consisting of 72 from each category of locations (e.g., an office), people and everyday objects (e.g., a telephone). Images of people included 36 neutral and 36 negative pictures (e.g., an injured person). All images of people were acquired from the International Affective Picture System (Lang, Bradley, & Cuthbert, 2005) and the Nencki Affective Picture System (Marchewka, Zurawski, Jednoróg, & Grabowska, 2013). We attempted to control for potential differences across stimuli by matching negative and neutral images of people. That is, we selected images that only displayed the head and shoulders of a person (see Figure 1a). Images of locations were acquired from the Internet and objects taken from a database of images used in previous research (Horner & Henson, 2008).

#### Procedure

Participants performed a single session involving encoding and test. At encoding, participants were presented with 36 ‘events’ (18 neutral and 18 negative) with each event including a location, person and object (Figure 1a). For negative events, only images of people were negative in valence, while the location and object were both neutral. Events were randomly generated across participants. During a single encoding trial, all three elements from an event (location, person, and object) were simultaneously presented on the screen. The three images were presented in a triangular configuration with one image presented in the middle of the screen above the center and the remaining two images presented to the left and right of center in the lower half of the screen. Placement of each element within events was randomized across trials. Each event was displayed for 6 s and was followed by fixation cross for a further 2 s. During each trial, participants were instructed to vividly imagine an event involving all three elements presented on screen and to be a creative as possible.

At test, participants completed a recognition and associative memory test for each single element and its paired associates (Figure 1a). That is, every association was tested in both directions resulting in a total of 216 tests trials (plus a further 216 new cue trials). Each trial started with the presentation of a cue image, which could either be a location, person or object. For example, on one trial the participant could be cued with an image of a person and shown six objects. Following interleaved trials from other events, the participant would be cued with the same person image and shown six locations to choose from. The six options on the alternative forced choice task always comprised (a) the correct association, (b) two foil images taken from events of the same emotional category as the correct response, and (c) three foil images taken from events of the opposite emotional category as the correct response. This was done to control for emotion at retrieval with all retrieval trials including three neutral and three negative options. A further 36 images from each element category were added as new items (including 18 neutral and 18 negative new person images). On presentation of the cue, participants were required to respond OLD or NEW via button press. If the cue was an old element, participants were then presented with six other images and participants were instructed to select the one that had been paired with the cue image at encoding. Participants were given a maximum of 10 s to make a response. Each trial ended with fixation cross that remained on screen for 2 s.

#### Analysis of memory coherence

The statistical dependency (see supplementary information for more details) between the retrieval of associations from the same event was assessed as in previous reports (Horner et al., 2015; Horner & Burgess, 2013, 2014). For each participant, we created 2 × 2 contingency tables for the retrieval of two elements when cued by the remaining within-event element (A_B_A_C_; e.g., cueing with a person to retrieve the associated location and object), as well as for retrieving an element when cued by its two associated elements (B_A_C_A_; e.g., retrieving a person when cued by the associated location and object). This resulted in six 2 × 2 tables per participant across each of the experimental conditions. To examine dependency, we took the proportion of events in which both associations were either correctly or incorrectly retrieved. We then averaged this measure across contingency tables for each condition.

We also created independent and dependent models of retrieval from each contingency table (see Table 1 for details on how these models were calculated). The independent model predicts the amount of dependency in relation to the participant’s mean level of performance for all associations across events, estimating the amount of statistical dependency expected if retrieval success for specific cue-test pairs (e.g., cue location, test person) is independent of retrieval success for other cue-test pairs (e.g., cue location, test object). The dependent model estimates retrieval performance for a given question adjusted by the mean performance over questions for that event (the episodic factor E). This allows us to predict the maximal dependency based on a participant’s overall performance, amount of guessing, and overall variance across all events.[Table-anchor tbl1]

### Results

#### Recognition memory performance

As each element from an event was presented twice at test (e.g., location served as a cue for both object and person associative test trials), recognition performance was assessed using a 2 × 2 × 3 repeated measures ANOVA (neutral vs. negative, first vs. second presentation, location vs. person vs. object cue; Figure 1b). Recognition memory performance was high across all conditions (see Table S1 for a full breakdown of hits and misses across conditions). Analysis of recognition memory performance (hits minus false alarms) showed no significant main effects of emotion (*F* [1, 16] = 2.79, *p* = .12, η_P_^2^ = 0.15), presentation (*F* [1, 16] = 1.07, *p* = .32, η_P_^2^ = 0.06) or cue type (*F* [2, 32] = 0.52, *p* = .60, η_P_^2^ = 0.03) and no interactions of Emotion × Cue Type (*F* [2, 32] = 0.65, *p* = .53, η_P_^2^ = 0.04), Emotion × Presentation (*F* [1, 16] = 0.77, *p* = .39, η_P_^2^ = 0.05), Presentation × Cue-Type (*F* [2, 32] = 0.78, *p* = .47, η_P_^2^ = 0.05) or Emotion × Presentation × Cue-Type (*F* [2, 32] = 0.19, *p* = .83, η_P_^2^ = 0.01).

#### Associative memory performance

As each event consisted of three separate associations between elements (location-object; person-location; object-person), we analyzed associative memory performance across these pairs (collapsed across test direction) using a 2 × 3 ANOVA (emotion; pair-type; Figure 1c). We saw a significant main effect of emotion reflecting better associative accuracy for neutral events compared to negative events (*F* [1, 16] = 17.10, *p* = .001, η_P_^2^ = 0.52). We also saw a main effect of pair type (*F* [2, 32] = 3.95, *p* < .05, η_P_^2^ = 0.20) with a tendency for better associative memory performance for object-location pairs compared to both person-object, *t*(16) = 2.29, *p* = .06, *d* = 0.55 and location-person pairs, *t*(16) = 1.84, *p* = .08, *d* = 0.44. Importantly, there was no interaction between emotion and pair-type (*F* [2, 32] = 0.01, *p* = .99, η_P_^2^ < 0.01) suggesting that the reduction in associative accuracy for negative events was consistent across all pair types (and not specific to pairs including a negative person element). Indeed, a direct comparison of associative accuracy for location-object pairs (comprising neutral elements for both neutral and negative events) showed reduced performance for negative compared to neutral events, *t*(16) = 4.39, *p* < .001, *d* = 1.06. This reduction in associative memory for negative events was also seen for person-object, *t*(16) = 3.51, *p* < .01, *d* = 0.85 and location-person pairs, *t*(16) = 3.03, *p* < .01, *d* = 0.73. In summary, there was a general reduction in associative memory performance for negative events and this reduction was evident across all pairs that formed part of the negative event.

#### Memory coherence

Coherence was assessed by constructing contingency tables for retrieving two elements when cued with the third element, and retrieving one element when cued by the other two elements across separate retrieval trials. We then calculated dependency (D) in the data by taking the proportion of events where elements were both correctly or incorrectly retrieved (see Methods and supplementary information for details). This dependency (D) was compared to the amount of dependency predicted if retrievals from the same event were completely independent (Di) or dependent (Dd; see Methods and supplementary information for details on how the models were constructed). We then compared the dependency in the data with both independent and dependent models separately for neutral and negative events (Figure 1d). Performing a 2 × 3 ANOVA (emotion: neutral or negative, dependency-measure: D, Di, Dd), we found a significant Emotion × Dependency-Measure interaction (*F* [2, 32] = 3.72, *p* < .05, η_P_^2^ = 0.20). Analyzing neutral events separately, we saw evidence of greater dependency in the data compared to the Independent model (D > Di, *t*[16] = 4.07, *p* = .001, *d* = 0.99) but no difference from the Dependent model, *t*(16) = 1.46, *p* = .16, *d* = 0.35.

For negative events, we again saw greater dependency in the data compared to the Independent model (D > Di, *t*(16) = 4.20, *p* = .001, *d* = 1.02) but this was less than that in the Dependent model (D < Dd, *t*(16) = 4.77, *p* < .001, *d* = 1.16). A direct comparison between neutral and negative events showed a greater decrease in dependency relative to the dependent model (D-Dd; *t*[16] = 4.47, *p* < .001, *d* = 1.08; no difference between neutral and negative events on the increase in dependency relative to the independent model; D-Di; *t*(16) = 0.48, *p* = .64, *d* = 0.11). Memory coherence was therefore reduced for negative relative to neutral events.

## Experiment 2

Experiment 1 showed that the presence of a negative element at encoding reduced subsequent memory for all within-event associations. Both neutral and negative events showed evidence of dependency when compared to the Independent model. However, dependency for negative events was lower than that predicted by the Dependent model, supported by an interaction between emotion and the dependency-measure, suggesting that negative elements at encoding reduce memory coherence. Previous studies have shown that events are not only stored as coherent representations when all elements are presented simultaneously (Horner & Burgess, 2013) but also when presented sequentially as overlapping paired associates (Horner et al., 2015; Horner & Burgess, 2014; Milivojevic, Vicente-Grabovetsky, & Doeller, 2015; Schlichting & Preston, 2015). We next examined how the presence of negative items might alter memory coherence when events were built up over a sequence of overlapping encoding trails, to separate the effects of associated negative elements from the effect of negative elements on the screen during encoding. In addition, we assessed whether coherence was further affected by negative pairs being encoded either early or late during the sequence of pairs forming an event.

Participants were required to learn events comprising three elements (location, person and object) presented sequentially as overlapping paired associates over a series of encoding trials (e.g., A-B, B-C, A-C) interleaved with trials from other events. Participants thus associated all within-event elements with each other despite never seeing all three at once (Horner & Burgess, 2014). In addition, we manipulated the order in which the events were encoded. For half of the events, the location-object pair was the first encoded (we refer to this condition as person-last). For the other half of the events, the location-object pair was the last pair encoded (we refer to this condition as person-first). As the negative element of an event was always the person element, negative events were therefore encoded with either the first or last study trial comprising a “pure-neutral” pair.

### Method

Experiment 2 was identical to Experiment 1 with the following changes:

#### Participants

A total of 26 participants (10 males) with a mean age of 24.16 years (*SD* = 3.16) were recruited from the university student population.

#### Procedure

**Figure 2 fig2:**
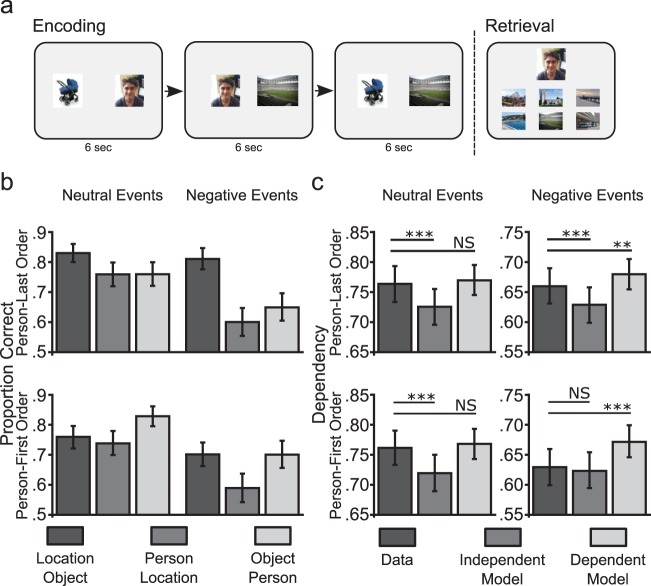
Memory for events encoded as overlapping pairs. (a) For Experiment 2, each event was encoded over three separate blocks (i.e., separated by encoding trials for other events). Events were either encoded with the location-object pair presented on the first encoding trial (person-last encoding order) or as the final encoding trial (person first encoding order). Associative memory was testing in a similar way to Experiment 1. (b) Associative memory performance for each encoded pair across neutral and negative events split by the person-last encoding order (upper panel, location-object studied first) and person-first encoding order (lower panel, object-person studied first). Note that the person image was always the negative element within negative events. (c) Dependency for the data and the independent and dependent models, across neutral and negative events split by person-last and person-first encoding orders. Note the overall decrease in dependency from neutral to negative events. Error bars represent standard error; NS = not significant. ** *p* ≤ .01. *** *p* < .001.

The materials used in Experiment 2 were exactly the same as Experiment 1. As in Experiment 1, participants encoded events consisting of a location, person and object. However, rather than simultaneously presenting all three items, participants viewed all event components as paired associates across three encoding blocks. The number of events at encoding was also increased to 72 (36 neutral and 36 negative events). For encoding, we presented events as paired associates across three blocks using two different encoding orders. For example, half of the events (18 neutral and 18 negative) were encoded using the order location-object, person-location, and object-person (person-last; Figure 2a). The other half of the events was presented using the order object-person, person-location, and location-object (person-first). Using these two orders allowed us to manipulate whether the first encoded pair of a negative event consisted of two neutral elements or a negative and neutral element. All paired associates were randomized within each encoding block. Each paired associate was presented for 6 s and was followed by a 2 s fixation inter trial interval. Participants were required to imagine the two items interacting in a meaningful way, and the overlapping nature of pairs across blocks was not mentioned. After encoding, participants completed the memory test (Figure 2a). The test was the same as described in Experiment 1 except that the recognition component was omitted (resulting in a total of 432 associative test trials). Therefore, on each trial participants were cued with one of the previously seen images and given a six alternative forced choice to try and remember the paired associate.[Fig-anchor fig2]

### Results

#### Associative memory

We analyzed associative memory performance for neutral and negative events for the three pair-types (location-object, person-location, object-person) under the two encoding orders (person-last; person-first) in a 2 × 2 × 3 ANOVA (emotion, encoding order, pair-type; Figure 2b). Memory for associations was generally worse for events that included a negative element (*F* [1, 25] = 60.07, *p* < .001, η_P_^2^ = 0.71). Performance also varied across pair-type (*F* [1, 50] = 16.89, *p* = .001, η_P_^2^ = 0.40) with better memory for the associations presented first compared to the second presented pair, which was always person-location (location-object in the person-last encoding order, *t*(25) = 5.16, *p* < .001, *d* = 1.01; person-object in person-first encoding order, *t*(25) = 4.99, *p* < .001, *d* = 0.98). Performance across pair-type also varied more for negative events (Emotion × Pair-Type interaction, *F* (1.62, 40.44) = 12.30, *p* < .001, η_P_^2^ = 0.33; Greenhouse-Giesser corrected). This interaction reflected worse memory for associations from negative events that involved a person element relative to location-object pairs. That is, there was a greater memory reduction for person-location relative to location-object pairs (*t*[25] = 4.77. *p* < .001, *d* = 0.94) and object-person pairs relative to object-location pairs, *t*(25) = 3.00, *p* < .01, 0.59 for negative compared to neutral event, a difference that was greater in the person-last encoding order (Order × Pair-Type, *F* [1.33, 33.21] = 13.86, *p* < .001, η_P_^2^ = 0.36).

We were interested to see whether negative items might influence memory for overlapping neutral items from the same event even when encoded in absence of the negative element (i.e., location-object pairs). While there was no difference in memory performance for the location-object pairs between neutral and negative events when studied under the person-last encoding order (when location-object is encoded before pairs involving negative elements, *t*(25) = 0.99, *p* = .33, *d* = 0.19), we found reduced associative accuracy for location-object pairs that were part of a negative events when studied during the person-first encoding order (when location-object is encoded last, *t*(25) = 2.64, *p* = .01, *d* = 0.52). Further, the difference in location-object accuracy from the person-last order to the person-first order showed a numerically greater reduction for negative compared to neutral events, though this was not statistically significant, *t*(25) = 1.72, *p* = .09, *d* = 0.34.

In summary, associative memory was consistently reduced by the presence of a negative element. We also found better associative accuracy for the initial pair from an event across both neutral and negative events, possibly due to event-related primacy effects. Interestingly, further analysis revealed that memory for the neutral pairs from a negative event was reduced when those items had been previously paired with a negative element (but not when they were subsequently paired with negative elements).

#### Memory coherence analysis

Dependency was calculated in the data (D) and compared to the dependencies Di and Dd as predicted by Independent and Dependent models (see Methods and supplementary information for details). A 2 × 2 × 3 repeated measures ANOVA (emotion; encoding-order; dependency-measure: D, Di, Dd) showed a significant three-way interaction (*F* [2, 50] = 3.43, *p* < .05, η_P_^2^ = 0.12). To further analyze this interaction, we performed separate 2 × 3 ANOVAs (encoding-order, dependency-measure) for neutral and negative events. For neutral events, we saw a significant main effect of dependency-measure (*F* [2, 50] = 24.46, *p* < .001, η_P_^2^ = 0.50) but no main effect of encoding order (*F* [1, 19] = .001, *p* = .98, η_P_^2^ < 0.01) or Order × Dependency-Measure interaction (*F* [2, 38] = 0.70, *p* = .50, η_P_^2^ < 0.01). Further analysis showed greater dependency (Figure 2c) for neutral events compared to the Independent model (D > Di, *t*[25] = 5.98, *p* < .001, *d* = 1.17) but no difference when compared to the Dependent model, *t*(25) = 1.56, *p* = .13, *d* = 0.31.

For negative events, this analysis showed a significant interaction of Encoding-Order × Dependency-Measure (*F* [2, 50] = 5.40, *p* < .01, η_P_^2^ = 0.18) and main effects of dependency-measure *F* [2, 50] = 30.76, *p* < .001, η_P_^2^ = 0.55; main effect of encoding-order *F* [1, 25] = 1.09, *p* = .31, η_P_^2^ = 0.04). Events encoded under the person-last order (pure-neutral pair presented first) showed evidence of increased dependency (Figure 2c) compared to the Independent model (D > Di, *t*[25] = 4.77, *p* = .001. *d* = 0.94) but also less dependency than the Dependent model (D < Dd, *t*[25] = 2.85, *p* < .01, *d* = 0.56). Negative events encoded using the person-first order (pure-neutral pair presented last) showed no evidence of dependency, with no difference between the data and Independent model, *t*[25] = 1.51, *p* = .14, *d* = 0.30 and significantly less dependency than the Dependent model (D < Di, *t*[25] = 4.41, *p* < .001, *d* = 0.86).

To directly compare dependency for negative events between the two encoding orders, we calculated dependency relative to the Independent model (D-Di). Importantly, this analysis showed greater dependency for the person-last encoding order, *t*(25) = 2.40, *p* < .05, *d* = 0.47, while a similar comparison for neutral events showed no significant difference between encoding orders, *t*(25) = 0.50, *p* = .62, *d* = 0.10; a difference reflected in the Emotion × Order × Dependency-Measure interaction. Similarly, we also performed a direct comparison on the dependency increase relative to the independent model (D-Di) between neutral and negative events. Supporting the reduction in dependency for negative events, this analysis found significantly greater dependency relative to the independent model for neutral compared to negative events when studied under the person-first encoding order, *t*(25) = 2.93, *p* < .01, *d* = 0.57. We saw no difference between neutral and negative studied under the person-last encoding order, *t*(25) = 0.54, *p* = .60, *d* = 0.12.

These results demonstrate that negative elements impair within-event dependency, and that this effect was particularly sensitive to the order in which elements were encoded. When the first two pairs within an event included a negative element (person-object; location-person, person-first encoding order), dependency did not differ from the independent model. In contrast, when the negative element was presented during the final two within-event encoding trails (i.e., the first trial included two neutral items, location-object, person-last encoding order), dependency was greater than the Independent model, although still less than predicted by the Dependent model (similar to the pattern of dependency seen for negative events in Experiment 1). Interestingly, we also saw a reduction in associative memory for neutral pairs from negative events when the previously encoded pairs from the same event included a negative item (i.e., person-first encoding order, Figure 2b).

## Experiment 3

As we have previously reported, the presence of negative stimuli during encoding can disrupt associative memory, possibly via hippocampal down-modulation, while memory for the negative content can be facilitated, possibly via amygdalar up-modulation (Bisby et al., 2016). We attempted to gain further insight into the results of Experiment 2 by adding a 24-hour delay between study and test to see whether postencoding processes such as consolidation might affect associative binding and memory coherence. Emotion is thought to modulate memory consolidation (McGaugh, 2000; Roozendaal & McGaugh, 2011), so we wanted to ascertain whether any effect of delay on associative memory or coherence was modulated by the presence of a negative element.

### Method

Experiment 3 was identical to Experiment 2 with the following changes:

#### Participants

A total of 27 participants (11 males) with a mean age of 23 years (*SD* = 2.56) were recruited from the university student population.

#### Procedure

The procedure was exactly the same as that used for Experiment 2 with the only difference being that test took place 24 hours after encoding.

### Results

#### Associative memory performance

**Figure 3 fig3:**
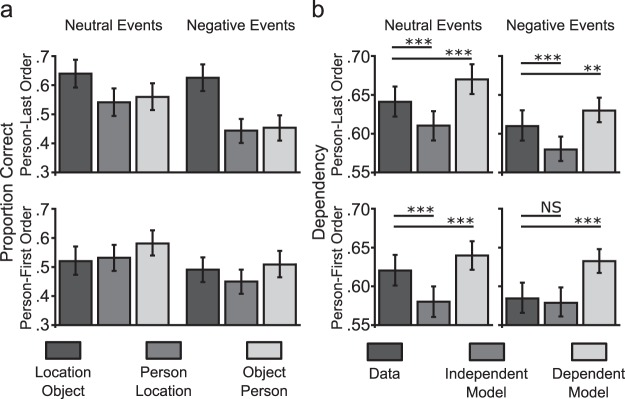
Memory for events following a 24hr delay. a, Associative memory performance across each pair type at encoding across neutral and negative events split by the person-last encoding order (location-object pair studied first) and person-first encoding order (object-person pair studied first). b, Dependency results for neutral and negative events following a 24-hour delay between encoding and test, split by the person-last and person-first encoding orders. Error bars represent standard error; NS = not significant. ** *p* < .01. *** *p* < .001.

We examined associative memory accuracy for the encoded pairs across neutral and negative events split by encoding order (Figure 3a and supplementary information for details). Performing a 2 × 2 × 3 ANOVA (emotion, encoding order, pair-type), we saw a similar pattern of performance to Experiment 2 (Figure 3a) with worse associative accuracy for negative events (*F* [1, 26] = 26.49, *p* < .001; η_P_^2^ = 0.51). Again, we found that performance varied across pair-type (*F* [2, 52] = 12.15, *p* < .001, η_P_^2^ = 0.32) and this was particularly evident for negative events (Emotion × Pair-Type, *F* [2, 52] = 5.99, *p* < .01; η_P_^2^ = 0.19) with worse memory for pairs that included the person element compared to location-object for negative compared to neutral events (person-location, *t*[26] = 2.38, *p* < .05, *d* = 0.46; object-person, *t*(26) = 3.57, *p* = .001, *d* = 0.69). Performance differences between pair-type were also greater during the person-last encoding order (Order × Pair-Type, *F* [2, 52] = 22.98, *p* < .001, η_P_^2^ = 0.47) with better memory for location-object pairs compared to person-location, *t*(26) = 8.43, *p* < .001, *d* = 1.62 and object-person, *t*(26) = 6.03, *p* < .001, *d* = 1.16. For the person-first encoding order, object-person accuracy was greater than person-location accuracy, *t*(26) = 2.62, *p* < .05, *d* = 0.50; all other p’s > 0.11. Similar to Experiment 2, associative accuracy was worse for pairs from a negative event compared to neutral events (person-location, *t*[26] = 4.16, *p* < .001, *d* = 0.80; object-person, *t*(26) = 4.30, *p* < .001, *d* = 0.83). However, there was no difference between neutral and negative events in accuracy for location-object pairs (the pair involving two neutral elements in both neutral and negative events, *t*(26) = 0.88, *p* = .39, *d* = 0.17).[Fig-anchor fig3]

#### Memory coherence analysis

We again calculated dependency in the data (D) and compared it to the dependencies Di and Dd as predicted by Independent and Dependent models (see Methods and supplementary information for details) by performing a 2 × 2 × 3 repeated measures ANOVA (emotion; encoding-order; dependency-measure). Consistent with Experiment 2, we found a three-way interaction (*F* [2, 52] = 5.56, *p* < .01, η_P_^2^ = 0.18) and therefore performed separate 2 × 3 ANOVAs on neutral and negative events.

Analysis of neutral events (see Figure 3b) showed a significant main effect of dependency-measure (*F* [2, 52] = 41.44, *p* < .001, η_P_^2^ = 0.61) and an effect of encoding order that approached significance (*F* [1, 26] = 4.15, *p* = .052, η_P_^2^ = 0.14; Order × Dependency-Measure interaction *p* > .7). Further analysis showed greater dependency when compared to the Independent model (D > Di, *t*[26] = 4.56, *p* < .001, *d* = 0.88) but also less dependency than the Dependent model (D < Dd, *t*[26] = 4.93, *p* < .001, *d* = 0.95; collapsed across encoding order). Importantly, there was no difference in dependency between encoding orders for neutral events relative to the independent model (i.e., in D-Di, *t*[26] = 0.52, *p* = .61, *d* = 0.10).

Analysis of negative events (see Figure 3b) showed a significant Encoding-Order × Dependency-Measure interaction (*F* [2, 52] = 7.45, *p* = .001, η_P_^2^ = 0.22) and a main effect of dependency-measure (*F* [2, 52] = 39.98, *p* < .001, η_P_^2^ = 0.61; main effect of encoding order *p* > .5). For events encoded using the person-last order (pure-neutral pair first), we saw significantly greater dependency compared to the Independent model (D > Di, *t*[26] = 4.26, *p* < .001, *d* = 0.82) and less dependency than the Dependent model (D < Dd, *t*[26] = 2.96, *p* < .01). In contrast, negative events encoded under the person-first order showed less dependency than the Dependent model (D < Dd, *t*[26] = 6.20, *p* < .001, *d* = 1.19) and no difference when compared to the independent model, *t*(26) = 0.31, *p* = .76, *d* = 0.06. A direct comparison of dependency relative to the independent model (D-Di) between encoding orders showed significantly greater dependency during the person-last encoding order, *t*(26) = 2.85, *p* < .01, *d* = 0.55. Similar to Experiment 2, there was more dependency relative to the independent model for neutral events studies under the person-first encoding order when compared to negative events (D-Di; *t*[26] = 3.07, *p* < .01, *d* = 0.59; no difference between neutral and negative events under the person-last encoding order, *t*(26) = 0.20, *p* = .85, *d* = 0.04).

We performed a further analysis to examine whether the 24-hour delay further contributed to effects of emotion on dependency (i.e., a direct comparison between Experiments 1 and 2). We therefore assessed the decrease in dependency relative to the Dependent model for neutral and negative events across Experiments 2 and 3 (D-Dd). A 2 × 2 × 2 mixed ANOVA (Experiment × Emotion × Order) with experiment added as a between participants factor showed a trend of a main effect of experiment (*F* [1, 51] = 3.58, *p* = .06, η_P_^2^ = 0.07; all other *p*’s > 0.43) with a greater decrease in dependency in Experiment 3 (i.e., following a 24-hour delay) suggesting that a 24 hour delay might reduce dependency. Importantly, a 24-hour delay did not interact with emotion on dependency (*p* > .12).

### A Computational Model

To explore potential neural mechanisms that might underlie the pattern of results observed in Experiment 2, we constructed a simple computational model of associative memory (Marr, 1971). We have previously shown that dependency for neutral events can emerge through a process of pattern completion within a model of hippocampal function (Horner et al., 2015). To examine the mechanisms that might support the pattern of results found here, we implemented a similar model.

As in Experiment 2, events were formed by encoding overlapping pairwise associations between separate neurons coding for individual elements within a fully recurrent attractor network. Encoding order and the associative structure of events were identical to Experiment 2. To account for overall behavioral performance, we assumed that the successful encoding of any given association was probabilistic. In addition, to model down-modulation of hippocampal synaptic plasticity by negative emotion, we assumed that the strength of successfully encoded pairwise associations was lower when a negative element was either presented or incidentally retrieved during encoding. Importantly, this does not prevent successfully encoded negative associations from being recalled, but does reduce pattern completion (and thus both performance and coherence) in negative events during retrieval. At retrieval, a single “cue” neuron was activated along with six “target” neurons that received partial activation to model the six-alternative forced choice task. Additional activity reflects inputs from recurrent synaptic currents, with retrieval of any element (excluding the cue) determined by a firing rate threshold. Overall performance was submitted to the statistical dependency analysis in the same way as behavioral data.

### Method

We simulate a simple network of *N* rate-coded neurons that are fully recurrently connected except for self-connections. The total input current to each neuron *I*_*total*_ is a combination of external and recurrent synaptic currents *I*_*ext*_ and *I*_*syn*_, respectively. Recurrent synaptic currents *I*_*syn*_ are equal to the product of synaptic weights *w*_*ij*_ and firing rates of connected neurons (Equation 1).

$$I_{total}=I_{ext}+I_{syn}I_{syn}=\sum_{j=1}^{N}w_{ij}r_{j}$$ 1

The firing rate *r*_*i*_ of each neuron is dictated by a threshold-linear activation function that converts the total input current *I*_*total*_ into a firing rate output, with a threshold of θ_*T*_ = 5nA and limited to a peak firing rate of *r*_*max*_ = 10Hz (see Equation 2, where [x]_+_ = 0 for x ≤ 0 and [x]_+_ = x for x > 0). All firing rates *r*_*i*_ and synaptic connections *w*_*ij*_ within the network are initially set to zero.

$$r_{i}=\left\{ \begin{array}{cc} {\left[I_{total}-\theta_{T}\right]}_{+} & {\text{for}\;I_{total}\leq 15nA}_{}\\ r_{\max} & \text{for}\;I_{total}>15nA \end{array}\right.$$ 2

Each element of an event is represented by a unique neuron. During encoding, neurons that represent the stimuli being presented in each trial receive a fixed amplitude synaptic current of *I*_*ext*_ = 15nA from an external source for a period of *t*_*enc*_ = 1000 ms. During this period, synaptic weights develop according to a Hebbian learning rule, that is, proportional to the product of pre- and post- synaptic firing rates and a learning rate *k*. In addition, we impose a postsynaptic firing rate threshold of θ_*p*_ = 7.5 Hz, analogous to the BCM learning rule, below which no synaptic weight change takes place (Equation 3; Bienenstock, Cooper, & Munro, 1982); and a hard limit of *w*_*max*_ = 1 on all synaptic weights.

$$\Delta w_{ij}=kr_{i}{\left[r_{j}-\theta_{p}\right]}_{+}$$ 3

To model variation in extracellular and intracellular conditions during encoding, we assume that learning is probabilistic, such that there is a probability *p*_*enc*_ = 0.65 that the learning rate will take a positive value *k* = *k*_*learn*_ and a probability 1 − *p*_*enc*_ that it will take a value of zero on any given encoding trial. The positive learning rate *k*_*learn*_ is a product of the learning rate for associations from neutral events *k*_*learn*_ = 1.6 × 10^−4^ and a modulation factor *m*. This modulation factor generally takes a value of *m* = 1, but is reduced to a value of *m* = 0.6 when any of the neurons that encode a negative element fire above a threshold rate θ_*neg*_ = 1Hz (Equation 4, where *H* represents the Heaviside function). Importantly, activity in neurons that encode a negative element can be generated either by external input (i.e., when an association including a negative element is being encoded), or by recurrent excitation (i.e., when a negative element is incidentally retrieved by association with one of the elements being encoded). The encoding order and resulting associative structures for the neutral and negative events are identical to Experiment 2.

$$\begin{array}{c} p\left(k=k_{learn}\right)=p_{enc}\\ p\left(k=0\right)=\left(1-p_{enc}\right)\\ k_{learn}=mk_{neut}\\ m=1-0.4H(\sum H(r_{i,neg}-\theta_{neg})) \end{array}$$ 4

During retrieval, neurons that represent the cued element receive a fixed amplitude synaptic current *I*_*ext*_ = 15nA from external sources for a period of *t*_*ret*_ = 1,000 ms, while neurons that represent the six forced choice target elements receive a constant current of *I*_*ext*_ = 5nA. Additional activity is generated by the recurrent synaptic currents, and the learning rate is set to zero (*k* = 0) to prevent further encoding. Behavioral performance is computed by setting a firing rate threshold of θ_*ret*_ = 5 Hz for ‘retrieval’ of any element (excluding the cue). Statistical dependency is then computed as described above. The retrieval order for each pairwise association for the neutral and negative event conditions was identical to Experiment 2 with the negative element presented during the last encoding trial (negative-last, corresponding to person-last in the behavioral experiments) or during the first encoding trial (negative-first, corresponding to person-first). Twenty-six simulations were performed, each containing 36 neutral and 36 negative events.

## Results

**Figure 4 fig4:**
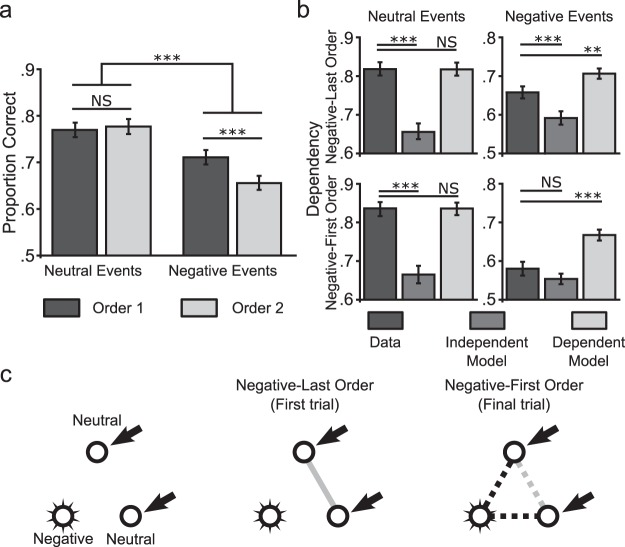
Model of associative learning and simulated results. (a) Performance and (b) Coherence (statistical dependency between retrievals from the same event) for each event type and encoding order (whether the negative element or person was presented last or first). (c) During encoding, negative events are constructed from three pairwise associations, one of which contains two neutral elements. Associative learning is reduced when the negative element (always a person) is active, so associations involving the negative element are weaker than those involving neutral events, reducing pattern completion, performance and dependency. If the neutral pair is presented after one or more negative-neutral pairs (the Negative-First Order), reactivation of the negative element can occur via the associations already learned to it, weakening the association formed between the neutral elements, further reducing pattern completion, performance and dependency.

In accordance with behavioral data, associative accuracy (Figure 4a) and dependency (Figure 4b) for negative events were both reduced in comparison to neutral events (irrespective of encoding order). Recurrent excitation passing through “strong” synaptic connections (i.e., those formed for neutral pairs) from the cue element via the nontarget element is sufficient to “retrieve” the target element, even when the direct association between cue and target elements has not been formed. This increases both overall performance, as associations can be recalled even when they were not successfully encoded; and dependency, as the likelihood of retrieving all three associations when only two have been successfully encoded is increased. Conversely, recurrent excitation passing through “weak” synaptic connections (e.g., those formed for negative pairs) from the cue element via the nontarget element is not sufficient to ‘retrieve’ the target element in the absence of a direct association between cue and target elements.[Fig-anchor fig4]

Similar to Experiment 2, negative events where the neutral pair was presented first (the order in which the negative element or person was presented last) showed increased associative accuracy (Figure 4a) and dependency (Figure 4b) compared to negative events where the neutral pair was presented last (the order in which the negative element or person was presented first). These differences can be accounted for by the model, due to incidental reactivation of negative elements at encoding. When the neutral pair from a negative event is presented first, a strong association between the neutral elements can be formed (see Figure 4c) and drive subsequent pattern completion. When the neutral pair from a negative event is presented last, however, there is a strong possibility that one or both of the neutral elements will have been associated with a negative element in previous learning trials. This leads to the reactivation of that negative element, and a subsequent reduction in the strength of the association formed between neutral elements that impairs pattern completion.

Hence, the model is able to reproduce each key feature of the experimental data provided that a single criterion is satisfied: the strength of learned connections between pairs of items that either include or cause the incidental retrieval of a negative element during encoding should be weaker than those between pairs of neutral items, such that they are sufficient to allow retrieval of that association but not support pattern completion (i.e., retrieval of the target element via reactivation of the third, nontarget element). The specific implementation and parameter values used here were chosen to ensure that this was the case, but any associative memory model that incorporated this property would produce similar results. This supports the hypothesis that each of the key experimental results can be accounted for by a single mechanism—a reduction in the strength of associations formed between pairs of items that include a negative element, or cause a negative element to be incidentally retrieved.

## General Discussion

A defining property of episodic memory is that events consisting of multiple elements are stored as coherent representations, so that episodic retrieval corresponds to holistic reexperience of all types of element (whether each is correctly remembered or not; Horner et al., 2015; Tulving, 1983). It has been argued that negative emotion will strengthen memory, resulting in a ‘general facilitation’ effect (Cahill & McGaugh, 1998; Rubin et al., 2008; Talarico et al., 2004). However, others have proposed that different aspects of memory will be affected in opposing ways (Brewin et al., 2010; Jacobs & Nadel, 1998) so that memory for the negative elements of an event may be strengthened but memory for associations between elements or between elements and their context may be weakened (Bisby et al., 2016; Madan et al., 2012).

Here we provide evidence that, while neutral events are bound together as coherent representations in memory, negative emotion can disrupt associative binding between event elements and weaken the coherence of those events. Across three experiments, the presence of a negative element within an event reduced both associative memory performance and the amount of statistical dependency between within-event elements. As demonstrated by our computational model, reduced coherence of negative events can be accounted for by weakened associative binding in the presence of negative elements, or the reactivation of their memory traces, which impairs pattern completion and thus the coherence of the event as experienced in holistic memory retrieval.

In accordance with previous findings (Horner et al., 2015; Horner & Burgess, 2013, 2014), retrieval of neutral events showed coherence (i.e., retrieval success for associations from the same event were statistically related), supporting the idea that episodic memories are unitary and retrieved holistically (Tulving, 1983). This coherence was present irrespective of whether events were encoded simultaneously or as overlapping pairs. It should be noted that some of the coherence seen when events are encoded simultaneously could be driven by variations in encoding strength between events (e.g., reflecting attention), but this is unlikely to be the case for sequentially presented events.

Negative events showed reduced associative accuracy and less coherence compared to neutral events. These reductions were observed when events were presented simultaneously or as a sequence of overlapping pairs. Successful encoding of all within-event pairs supports a coherent associative structure and enables pattern completion of all event elements at retrieval irrespective of which is the cue or the target, increasing coherence. We assume that the presence of a negative element at encoding could reduce the formation of associations with other elements presented in conjunction (Bisby & Burgess, 2014; Kensinger & Schacter, 2006; Madan et al., 2012; Mather & Knight, 2008; Rimmele et al., 2011; Touryan et al., 2007), decreasing associative memory performance and coherence. Our computational model verified this assumption as a potentially valid explanation of our data. Given the important role of the hippocampus in associative/relational binding (Davachi, 2006; Eichenbaum et al., 2007; O’Keefe & Nadel, 1978) and within-event dependency (Horner et al., 2015), our results are consistent with reports that negative emotion might down-modulate hippocampal processing to impair associative memory formation (Bisby et al., 2016).

Dependency for negative events showed a greater reduction when the initial studied pair included a negative element, highlighting the significance of the order in which events were studied. A decrease in our measure of dependency reflects a weaker relationship between retrieval success for different associations from the same event (i.e., the success of retrieving one element from an event did not predict retrieval of other elements from the same event). Thus, reduced dependency when the initial encoded pair included a negative element highlights a potential lack of pattern completion to support holistic retrieval of all event elements. Negative events were therefore encoded as less coherent representations than neutral events, and this reduction was accentuated by encoding order.

Interestingly, it has been shown that participants are worse at learning novel associations between a negative item and its screen location when the negative item has previously been encoded (Nashiro, Sakaki, Huffman, & Mather, 2013). Further, the amygdala is thought to prevent memory updating when negative items are later reencountered and that novel learning requires its inhibition via orbitofrontal areas (Sakaki, Niki, & Mather, 2011). The combination of these findings and our own demonstrate how encoded negative items can further disrupt encoding on subsequent learning trials whether the negative item is later presented or when items that have previously been associated with a negative item are presented.

For neutral events, pattern completion could facilitate memory performance and coherence even when two elements are only weakly associated, retrieval being boosted by activity passing though indirect connections via the third element. This would strengthen weak associations and support inference and integration of overlapping information (Milivojevic et al., 2015; Zeithamova, Dominick, & Preston, 2012). These pattern completion processes can also account for differences in coherence across encoding orders for negative events. Comparable accuracy for location-object pairs across neutral and negative events when presented on the first encoding trial (person-last encoding order) suggest a strong association was formed. This strong association could provide a basis for pattern completion during subsequent encoding trials or during test, aiding performance and coherence, as shown in our model. In contrast, a negative element on the first encoding trial (person-first encoding order) would mean no strong association would be present on subsequent encoding or retrieval trials.

When a negative element was presented on the initial encoding trials of the event (as in the person-first encoding order), formation of these negative associations can disrupt encoding of subsequent overlapping pairs. Subsequent presentation of neutral elements associated to the negative element may result in activation of the negative element which would disrupt the formation of new associations, even in the absence of the negative element. This would explain the reduced associative accuracy for neutral pairs from negative events during the person-first encoding order. The formation of associations/relations between the emotional properties and items could be supported by the amygdala, consistent with an emotional binding hypothesis (Bisby et al., 2016; Yonelinas & Ritchey, 2015).

This is the first study to examine episodic memory coherence following a prolonged delay. Interestingly, coherence for neutral events was slightly (though not significantly) reduced after 24 hours, suggesting that some of the important associations required to form, and maintain, coherent representations might be weakened or lost over time. The pattern of associative memory and coherence results for negative events were replicated across delays with a similar pattern observed whether memory was tested immediately or following a 24 hour delay. It is well established that negative emotion can influence memory during both encoding (Dolcos, LaBar, & Cabeza, 2004; Kensinger & Corkin, 2004) and consolidation (LaBar & Cabeza, 2006; McGaugh, 2004). However, many of these reports have demonstrated enhanced memory for individual emotional items or the subjective feelings attached to them (Cahill & McGaugh, 1995; Sharot et al., 2004). In contrast, our findings support the view that the disruptive nature of negative emotion on associative binding can occur during encoding (or retrieval), but perhaps does not affect consolidation.

While we provide clear evidence that associative binding was impaired by negative emotion, we did not expect that memory for the individual elements themselves should be reduced, indeed memory for individual items is often enhanced by negative emotional content (Bisby & Burgess, 2014; Cahill & McGaugh, 1995; Sharot et al., 2004; Sharot & Yonelinas, 2008; Yonelinas & Ritchey, 2015). Although we only directly assessed item recognition in Experiment 1, our results show comparable memory performance across neutral and negative conditions. This is consistent with the view that the negative emotion specifically impairs associations, possibly through disrupting hippocampal function, while memory for individual items could be supported by modality-specific neocortical regions (Aggleton & Brown, 1999; Eichenbaum et al., 2007; Marr, 1971; McClelland et al., 1995) and their affective properties associated to them via the amygdala (LeDoux, 2000; Paz, Pelletier, Bauer, & Paré, 2006; Wang et al., 2014).

The precise mechanisms that might support the up- and down-modulation of distinct memory representations during a negative event are unclear. However, an arousal-biased competition model (Mather & Sutherland, 2011) proposes that observed memory enhancements and impairments are generated via competition of limited mental resources for encoded information. Within this account, arousal is thought to bias processing toward high priority representations at a cost of low priority representations, changes which could be supported by complex interactions between glutamate and norepinephrine (Mather, Clewett, Sakaki, & Harley, 2016). While this highlights a potential mechanism that could contribute to our pattern of results, this competitive mechanism would require extension to explain the reduction in associative memory for pairs of neutral items encoded after related negative associations (Experiments 2 and 3), the absence of an inverse relationship between memory for negative and neutral items in Experiment 1, and the reduction in associative memory seen between pairs of negative items in previous studies (Bisby et al., 2016; Bisby & Burgess, 2014). Further studies will be required to fully elucidate the complex mechanisms supporting impaired associative memory for negative events.

The salience of emotional items is likely to attract greater processing due to their attentional capture and distinctiveness (Talmi, 2013), but we do not think that these attributes can fully account for observed reductions in associative memory. Studies demonstrate similar reductions in associative memory for emotional words, even when word stimuli are well-matched across numerous dimensions (Madan et al., 2012) and this pattern of behavior is mirrored when using emotional pictures (Bisby & Burgess, 2014). Item memory was unaffected by emotion suggesting that attentional processing between emotional categories did not contribute to the pattern of associate memory and dependency. Further evidence against an attentional explanation can be drawn from Experiment 2, in which associative memory was reduced for location-object pairs when each item had previously been paired with a negative item (which was no longer on the screen to distract attention). In a series of experiments, we have also shown that when participants encode neutral and negative item-context pairings on background contexts that predict wither safety or threat of shock, the threatening contexts have little effect on item memory but impair the association between the neutral item and its context (Bisby & Burgess, 2014). Further, when participants encode negative-negative item pairs (both of which should capture attention), associative memory is still disrupted compared to neutral-neutral item pairs (Bisby & Burgess, 2014; Bisby et al., 2016). Further experiments could attempt to dissociate salience from emotion within the current experimental design by replacing emotional items with nonemotional salient items (e.g., “oddballs”; Strange, Hurlemann, & Dolan, 2003) to see whether they also affect associative memory in a similar way. Taken together, while attention and distinctiveness surely play an important role in emotional memory alterations, our results suggest that they cannot fully account for the pattern of our results.

Our findings have important clinical implications for the way in which negative events might contribute to memory disturbances, as seen in disorders such as posttraumatic stress disorder (Brewin, 2003; PTSD). The debate between “general facilitation” and “dual representation” accounts of the effect of emotion on memory extends to a debate concerning whether a traumatic event will lead to a generally strengthened memory or a fragmented memory comprising some very strong elements and some absent or weak elements (cf. Brewin et al., 2010; Rubin et al., 2008). Here, we demonstrate that experiencing mildly negative events in healthy volunteers reduced the coherence of episodic memories. This fragmented associative structure for negative events reduces the likelihood that a partial cue would trigger holistic retrieval via pattern completion. Thus our findings support the view that traumatic memories, like the mildly negative emotional memories used here, might be fragmented rather than simply strengthened relative to neutral memories. It is important to note that providing more information about the event may strengthen memory for the negative content by increasing the number of retrieval cues (Pearson, 2012; Pearson, Ross, & Webster, 2012), as might ongoing reactivation or rumination concerning the event (Berntsen, Staugaard, & Sørensen, 2013; Rubin et al., 2008), facilitating subsequent retrieval via sensory cues (Kleim, Ehring, & Ehlers, 2012). However, a dual representation account adds to this understanding by proposing how altered associative processing at encoding can also contribute to memory disruptions.

Our findings have potential therapeutic implications. They highlight the importance of the formation of associations between the negative element of an event and the surrounding neutral elements or context. This suggests that an important process in recovery of healthy memory function could be the formation of strong associations between the negative content and the neutral context of the event. However, our findings also indicate that the continued presence of negative emotion might hinder the formation of new associations. Trauma-focused psychological therapies such as imagery rescripting (Hackmann, 1998) and eye movement desensitization and reprocessing (Shapiro, 2001; EMDR) aim at revisiting the negative information while ameliorating its negative emotional impact. These techniques often require patients to elaborate on the negative imagery, thus associating them to appropriate neutral contextual information (such as when and where it happened). The attenuation of symptoms might be understood in terms of the mechanisms of episodic memory formation studied here and the strengthening of weak connections to reestablish coherent episodic memories, as predicted by a dual representation framework (Brewin et al., 2010).

In conclusion, we provide new evidence that negative events can disrupt associative binding and the coherence of single representations in memory. While neutral events were consistently found to be bound together as single representations in memory, negative emotion disrupts associative binding between event elements, resulting in a weakened associative structure. The presence of a negative element consistently resulted in a decrease in statistical dependency between within-event elements. Overall, our data and computational model demonstrate that reduced coherence in memory for negative events can be accounted for by weakened associative binding in the presence of negative elements, disrupting pattern completion processes that support holistic retrieval. These findings highlight the importance of associative/relational memory mechanisms in contributing to memory disturbances in PTSD and their treatment by therapeutic interventions.

### Code Availability

The code for the computation model used in this article is freely available on FigShare (http://dx.doi.org/10.6084/m9.figshare.5240752).

### Data Availability

The data presented in this article are freely available on FigShare (http://dx.doi.org/10.6084/m9.figshare.5240749).

## Supplementary Material

10.1037/xge0000356.supp

## Figures and Tables

**Table 1 tbl1:** Contingency Tables for Independent and Dependent Models Giving the Frequency (Over Events) of the Four Combinations of Correct or Incorrect Retrievals of Elements B and C When Cued With Element A

Retrieval of element (C)	Retrieval of element (B)	
	Correct (*P*_*AB*_)	Incorrect (1 − *P*_*AB*_)
Independent model		
Correct (*P*_*AC*_)	∑_*i*=1_^*N*^*P*_*AB*_*P*_*AC*_	∑_*i*=1_^*N*^*P*_*AC*_(1 − *P*_*AB*_)
Incorrect (1 − *P*_*AC*_)	∑_*i*=1_^*N*^*P*_*AB*_(1 − *P*_*AC*_)	∑_*i*=1_^*N*^(1 − *P*_*AB*_)(1 − *P*_*AC*_)
Dependent model		
Correct (*P*_*AC*_)	∑_*i*=1_^*N*^*Ṕ*^*i*^_*AB*_*Ṕ*^*i*^_*AC*_	∑_*i*=1_^*N*^*Ṕ*^*i*^_*AC*_(1 − *Ṕ*^*i*^_*AB*_)
Incorrect (1 − *P*_*AC*_)	∑_*i*=1_^*N*^*Ṕ*^*i*^_*AB*_(1 − *Ṕ*^*i*^_*AC*_)	∑_*i*=1_^*N*^(1 − *Ṕ*^*i*^_*AB*_)(1 − *Ṕ*^*i*^_*AC*_)
*Note.* Dependent model replaces the probability of correctly recalling B when cued with A (across all events; *P*_*AB*_) with *Ṕ*^*i*^_*AB*_ = *E*^*i*^_*AB*_ (*P*_AB_ – *P*_*G*_/c) + *P*_*G*_/*c* where the episodic factor *E*_*AB*_^*i*^ reflects performance on event *i* relative to other events (based on retrievals other than B and C cued by A), *P*_*G*_ is the probability of guessing, and *c* = 6 is the number of choices in the test trial. *P*_*AC*_ and *Ṕ*_*AC*_ defined similarly. The dependency model equates to the independent model if the episodic factors are set to 1.
