# Supplementary material for: Negative Emotional Content Disrupts the Coherence of Episodic Memories
Source: J Exp Psychol Gen. 2017 Sep 14;147(2):243–56. doi: 10.1037/xge0000356 (PMC5784934; doi:10.1037/xge0000356)
Supplement: Supplementary file 1 [file zfr999172952so1.docx]

**Supplemental Materials**

**Negative Emotional Content Disrupts the Coherence of Episodic Memories**

**by J. A. Bisby et al., 2017, *Journal of Experimental Psychology: General***

**http://dx.doi.org/10.1037/xge0000356**

**Supplementary Results:**

| **Table S1.** Proportion correct (SD) for recognition hits and false alarms during Experiment 1 across neutral and negative events for each cue presentation. Note that new locations and objects cannot be split by emotion. | | | | | | |
| --- | --- | --- | --- | --- | --- | --- |
|  | Presentation | | | | | |
| Cue type | First | | | Second | | |
|  | Neutral | | Negative | Neutral | | Negative |
| Recognition |  | | |  | | |
| Location | .91(.08) | .87(.13) | | .97(.03) | | .95(.06) |
| Person | .89(.07) | | .86(.11) | .94(.07) | .92(.10) | |
| Object | .90(.09) | | .89(.11) | .95(.06) | | .95(.05) |
|  |  | | |  | | |
| False alarms |  | | |  | | |
| Location | .02(.03) | | | .06(.06) | | |
| Person | .02(.03) | | .02(.03) | .05(.08) | | .04(.07) |
| Object | .01(.03) | | | .07(.09) | | |
|  |  | | |  | | |

| **Table S2.** Proportion correct (SD) associative memory performance during Experiment 1 across neutral and negative events for each cue and retrieval type. | | | | | | |
| --- | --- | --- | --- | --- | --- | --- |
|  | Retrieval type | | | | | |
| Cue type | Location | | Person | | Object | |
|  | Neutral | Negative | Neutral | Negative | Neutral | Negative |
| Recognition Hits |  | |  | |  | |
| Location | n/a | | .92(.07) | .91(.06) | .96(.06) | .92(.08) |
| Person | .92(.07) | .89(.12) | n/a | | .92(.07) | .89(.10) |
| Object | .92(.09) | .92(.09) | .93(.06) | .91(.07) | n/a | |
|  |  | |  | |  | |
|  |  | |  | |  | |

| **Table S3.** Proportion correct (SD) for associative memory performance during Experiment 2 across neutral and negative events for each cue and retrieval type split by the order in which they were encoded. | | | | | | |
| --- | --- | --- | --- | --- | --- | --- |
|  | Retrieval type | | | | | |
| Cue type | Location | | Person | | Object | |
|  | Neutral | Negative | Neutral | Negative | Neutral | Negative |
| Encoding Order 1 |  | |  | |  | |
| Location | n/a | | .76(.21) | .61(.26) | .84(.16) | .83(.15) |
| Person | .78(.19) | .60(.26) | n/a | | .77(.22) | .63(.27) |
| Object | .83(.16) | .91(.09) | .79(.19) | .67(.24) | n/a | |
| Encoding Order 2 |  | |  | |  | |
| Location | n/a | | .74(.20) | .60(.28) | .76(.21) | .73(.21) |
| Person | .77(.21) | .59(.25) | n/a | | .85(.14) | .71(.24) |
| Object | .80(.17) | .71(.21) | .86(.16) | .72(.22) | n/a | |
|  |  | |  | |  | |

| **Table S4.** Proportion correct (SD) for associative memory performance during Experiment 3 across neutral and negative events for each cue and retrieval type split by the order in which they were encoded. | | | | | | |
| --- | --- | --- | --- | --- | --- | --- |
|  | Retrieval type | | | | | |
| Cue type | Location | | Person | | Object | |
|  | Neutral | Negative | Neutral | Negative | Neutral | Negative |
| Encoding Order 1 |  | |  | |  | |
| Location | n/a | | .53(.26) | .47 (.25) | .65 (.24) | .64 (.26) |
| Person | .54 (.27) | .42 (.21) | n/a | | .56 (.25) | .45 (.24) |
| Object | .63 (.27) | .63 (.25) | .56 (.25) | .47 (.24) | n/a | |
| Encoding Order 2 |  | |  | |  | |
| Location | n/a | | .53 (.23) | .43 (.22) | .52 (.25) | .51 (.23) |
| Person | .52 (.27) | .47 (.22) | n/a | | .57 (.26) | .53 (.24) |
| Object | .52 (.26) | .47 (.25) | .59 (.22) | .49 (.25) | n/a | |
|  |  | |  | |  | |

**Supplementary Methods:**

**Memory coherence analysis.** To assess memory coherence, we created a measure of statistical dependency. Contingency tables were constructed for each participant for (1) retrieving two elements of an event across separate trials when cued by the other element of the same event (the AbAc analysis) and (2) retrieving one element from an event across separate trials when cued by the other two elements from the event. The tables therefore show how the retrieval of one association from an individual event depends on the retrieval of another association from the same event. Each analysis is performed for (1) the location as a cue – the location AbAc analysis, (2) the location as a retrieval target – the location BaBc analysis, (3) the person as a cue – the person AbAc analysis, (4) the person as a retrieval target – the person BaBc analysis, (5) the object as a cue – the object AbAc analysis, and (6) the object as a retrieval target – the object BaBc analysis. The resulting contingency tables therefore always relate to a common cue or retrieval target element and assess performance of the two overlapping associations (for example, location-person and location-object).

We also created Independent and Dependent models for each contingency table. The models are constructed to provide an estimate for the amount of dependency for a participant given various factors, including their general level of performance and guessing. The Independent model provides an estimate of the amount of dependency expected if retrieval of all elements from an event are independent. The Independent model is calculated by multiplying the probabilities of separately retrieving two elements. For example, if assessing the amount of dependency for retrieving the person and object when cued by the location, for the cell of the contingency table referring to both associations correctly retrieved we multiply the probability of retrieving the person when cued by the location ($P_{AB}$) across all events ($N$) with the probability of retrieving the object when cued by the location ($P_{AC}$) across all events.

The Dependent model expands upon the Independent model by including an episodic factor ($E^{i}$) that varies across events. This factor weights performance for an event $i$ by the extent to which performance for that event across multiple retrieval trials differs from performance across all events. For example, when retrieving B when cued by A for event $i$:

$$E_{AB}^{i}=\left( T_{BA}^{i}+T_{BC}^{i}+T_{CA}^{i}+T_{CB}^{i} \right)/\left( P_{BA}+P_{BC}+P_{CA}+P_{CB} \right)$$

whereby, $T_{BA}^{i}=1$ if the participant correctly retrieves A when cued by B for event $i$ (otherwise, $T_{BA}^{i}=0$), and similarly for $T_{BC}^{i}$ and so on, whereby $T_{BC}^{i}$ relates to the specific retrieval ($T$). The probability of correctly retrieving an association from event $i$ is weighted by the episodic factor for that event, that is, $P_{AB}$ becomes ${P'}_{AB}^{i}=E_{AB}^{i}P_{AB}$. In addition, the dependent model also controls for the level of guessing so that $E^{i}$ weights the probability of intentional correct retrieval but not the probability of guessing correctly. The dependent model therefore follows the independent model, with $P_{AB}^{i}$ (and also $P_{AC}^{i}$ and so on for all other associations) replaced by:

$${P'}_{AB}^{i}=E_{AB}^{i}\left( P_{AB}-P_{G}/c \right)+P_{G}/c$$

whereby $P_{G}$ is the proportion of guesses, of which $P_{G}/c$ will be correct in a $c$-way forced alternative choice cued recognition ($c=6$ in the experiments presented here). $P^{G}$ is estimated as $c/\left( c-1 \right)$ times the proportion of errors. For the Independent model, $E^{i}$ is set to 1 across all events.

We therefore build contingency tables for each participant for the data, Independent and Dependent models cross six different analyses (analysis type – AbAc versus BaCa and element type – location, person and object). For each table, we calculate a measure of dependency based on the proportion of events where both associations are retrieved correctly or incorrectly, where 1 = full dependence and 0.5 = full independence.
